# Hidden in plain sight: verbal declarative memory effects of exercise are eight times larger than aggregate estimates suggest

**DOI:** 10.3389/fnhum.2026.1902252

**Published:** 2026-09-03

**Authors:** Mengxuan Tian, Sainyu Guo

**Affiliations:** 1 School of Foreign Languages, Shandong Technology and Business University, Yantai, China; 2 Physical Education Department, Shandong Technology and Business University, Yantai, China

**Keywords:** acute exercise, cognitive enhancement, dose–response, exercise prescription, meta-meta-analysis, verbal declarative memory

## Abstract

**Objective:**

A recent meta-meta-analysis concluded that the average effect of exercise on memory is near zero after correcting for publication bias (SMD = 0.027). We tested whether this null finding reflects excessive aggregation rather than a genuine absence of effect.

**Methods:**

We conducted a focused reanalysis a publicly available meta-meta-analytic database, applying a theoretically motivated outcome reclassification protocol to isolate verbal declarative memory (VDM). From 2,239 effect-size estimates spanning 215 meta-analyses, *k* = 37 VDM-specific estimates were synthesized using a random-effects model. Publication bias was assessed via Egger’s test, PET-PEESE, and trim-and-fill.

**Results:**

The pooled effect was *g* = 0.216 [95% CI (0.075, 0.357), *p* = 0.003], approximately eight times larger than the aggregate estimate of 0.027. Moderate heterogeneity was observed (*I*^2^ = 56.1%), with no significant funnel plot asymmetry (*p* = 0.458). Aerobic exercise produced the most robust benefit (*g* = 0.229, *k* = 27).

**Conclusion:**

The null aggregate memory effect masks a meaningful signal for verbal declarative memory. The findings support domain-specific exercise prescriptions: for hippocampus-dependent verbal memory, moderate-intensity aerobic exercise represents an evidence-supported strategy for enhancing retention.

## 1 Introduction

The proposition that physical exercise can enhance cognitive function has attracted enormous research attention over the past two decades. Hundreds of randomized controlled trials (RCTs) and dozens of meta-analyses have examined whether acute and chronic exercise improve performance across attention, executive function, processing speed, and memory (Stillman et al., 2020; El-Sayes et al., 2019). Convergent meta-analytic evidence now supports a small but reliable overall benefit. A recent umbrella review of 133 systematic reviews encompassing 2,724 unique RCTs and 258,279 participants confirmed a significant overall effect of exercise on cognition, with memory-specific effects estimated at SMD = 0.26 (Singh et al., 2025). Chang et al. (2025), in a meta-review of 30 systematic reviews with 383 primary studies and 18,347 participants, independently corroborated this estimate (memory SMD = 0.23). Mavilidi et al. (2025), synthesizing 171 chronic and 68 acute studies with 48,625 participants across the lifespan, demonstrated that moderate-to-vigorous intensity physical activity produced the largest cognitive benefits (*g* = 0.63–0.71). This convergent evidence base has fueled optimistic messaging in public health and educational policy, with exercise increasingly promoted as a cognitive enhancer with applications in classrooms, workplaces, and clinical settings (World Health Organization, 2022; Herold et al., 2019).

A landmark meta-meta-analysis compiled 2,239 effect-size estimates from 215 meta-analyses of exercise-cognition RCTs and reached a sobering conclusion: after correcting for publication bias, the average effect of exercise on memory is effectively zero [SMD = 0.027, 95% credible interval (0.000, 0.227); Bartos et al., 2025]. Recent methodological reanalyzes in other domains have demonstrated that meta-analytic conclusions can be sensitive to specification and classification decisions (Sorjonen and Melin, (2025),), reinforcing the need to scrutinize outcome category definitions in exercise-cognition synthesis. This finding stands in marked contrast to individual behavioral experiments that have consistently reported positive effects using rigorous within-subject designs. Loprinzi et al. (2023), in the most methodologically rigorous dose–response investigation to date (*N* = 59, 12 laboratory visits, within-subjects crossover), demonstrated that both moderate- and vigorous-intensity cycling enhanced 24-h delayed recall of word lists relative to seated rest, with higher baseline aerobic fitness predicting larger benefits. Makepeace and Craig (2024) replicated the intensity-dependent effect in an ecologically valid field setting with endurance runners. Morita et al. (2024) extended the retention window to 8 weeks with a single pre-encoding bout of moderate-intensity aerobic exercise. Patrick et al. (2025) demonstrated that even a 20-min bout of moderate-intensity exercise improved free recall in young adults. Loprinzi and Patrick (2024) further showed that both 20- and 40-min moderate-intensity bouts were equally effective. These individual study findings, converging on a small-to-moderate positive effect for verbal episodic memory, appear irreconcilable with the aggregate null. How can exercise trigger the neurochemical machinery of memory formation elevating BDNF, cortisol, epinephrine, and catecholamines in a dose-dependent manner (Hotting et al., 2016; Zhang et al., 2025a; Zhang et al., 2025b) yet produce no detectable behavioral benefit on memory itself?

Memory is not a unitary cognitive construct. Declarative memory encompasses multiple dissociable subsystems, including verbal memory (word lists, stories, paired associates), visual memory (faces, scenes, object locations), and spatial memory (navigation, route learning), each engaging partially distinct neural circuitry (Squire, 2004; Eichenbaum, 2017). Verbal declarative memory (VDM) depends on coordinated activity between the hippocampus critical for the rapid encoding of arbitrary associations, such as word-meaning pairings and left-lateralized perisylvian language networks supporting semantic integration. This neuroanatomical profile distinguishes VDM from visuospatial memory, which recruits right-hemisphere parietal and parahippocampal regions. If exercise preferentially facilitates hippocampal function as the BDNF-mediated long-term potentiation (LTP) hypothesis predicts (El-Sayes et al., 2019; Lima et al., 2024) then its mnemonic benefits should be most pronounced for hippocampus-dependent memory subtypes rather than for memory as an undifferentiated whole. The null aggregate effect reported by Bartos et al. (2025) may therefore reflect the dilution of a genuine signal within verbal memory by null or negligible effects in non-hippocampal memory subtypes. Two recent meta-analyses provided direct evidence for this domain-specificity prediction. Qazi et al. (2024), synthesizing 42 experiments, demonstrated that exercise before encoding significantly improved free recall (*d* = 0.40) but not recognition (*d* = −0.06), while exercise after encoding improved recognition (*d* = 0.62) more than free recall (*d* = 0.19) a double dissociation implicating distinct encoding and consolidation mechanisms that are differentially sensitive to exercise timing. Schmid et al. (2024), in a meta-analysis of children, corroborated this dissociation: acute and chronic physical activity enhanced free recall (*g* = 0.56) and cued recall (*g* = 0.67), whereas recognition memory was not significantly improved (*g* = 0.06).

Baumgartner et al. (2024) reported null effects of high-intensity resistance exercise on relational memory in a counterbalanced crossover design (*N* = 36), with acute increases in BDNF and lactate failing to predict memory changes. Loprinzi and Caplan (2025) found that acute exercise did not enhance mnemonic discrimination a hippocampal DG-CA3 pattern separation task that taxes computations distinct from those supporting associative recall. Zhai et al. (2025), published in Psychology of Sport and Exercise, showed that acute aerobic exercise enhanced memory encoding but not consolidation in individuals with methamphetamine use disorder. Wang et al. (2024) extended this work to the neural level, demonstrating that physical activity-related improvements in verbal memory were mediated by individual differences in resting-state brain activity. This accumulating pattern robust effects on verbal free recall, weaker or null effects on recognition, discrimination, and relational memory is precisely what the domain-specificity hypothesis predicts, yet no meta-analysis has formally isolated verbal declarative memory from the broader memory category to quantify its specific effect size.

Beyond the question of memory specificity, a second critical gap concerns the dose–response relationship between exercise intensity and cognitive outcomes. Recent large-scale meta-analyses have begun to map this landscape. Yuan et al. (2024) employed a Bayesian network meta-analysis to identify an optimal exercise dose of approximately 650 METs-minutes per week (SMD = 0.691) for global cognition, with benefits attenuating beyond 1,000 METs-minutes per week a pattern consistent with the inverted-U arousal-performance relationship predicted by the Yerkes-Dodson law. Ye et al. (2024) demonstrated that optimal exercise parameters vary considerably by cognitive subdomain: working memory benefits most from 20 to 45 min sessions at moderate-to-progressive intensity, whereas inhibitory control is optimally enhanced by low-intensity exercise. Liu et al. (2024) reported that HIIT improved memory with an SMD of 0.21. On the mechanistic side, Rodriguez-Gutierrez et al. (2024) demonstrated in a network meta-analysis of 22 RCTs that HIIT was the most effective modality for acutely increasing peripheral BDNF (SMD = 1.49 vs. control), while Li et al. (2024) confirmed that exercise intensity is the primary driver of CREB phosphorylation and downstream BDNF synthesis. Mielniczek and Aune (2024) further showed that BDNF responses to HIIT are protocol-dependent, with significant individual variability. Herold et al. (2019) issued an explicit call for the field to transition from whether exercise benefits cognition to what exercise parameters optimize which cognitive outcomes, a call echoed by Bartos et al. (2025) in their conclusion advocating targeted, population- and intervention-specific recommendations. Critically, however, the dose–response relationship between exercise intensity and VDM remains uncharacterized at the meta-analytic level, primarily because primary meta-analyses have overwhelmingly pooled effect sizes across intensity levels without reporting intensity-stratified estimates (Loprinzi et al., 2021). We anticipated that exercise intensity could not be directly examined as a moderator in the present analysis for this reason, but we identify this dose–response gap as a primary motivation for our domain-specificity approach and a priority for future primary research.

Drawing on the domain-specificity framework outlined above which integrates the Yerkes-Dodson arousal law, the BDNF-mediated arousal-consolidation hypothesis, and the complementary learning systems account of hippocampal-neocortical memory dissociation the present study addresses these gaps through a focused reanalysis of the publicly available Bartos et al. (2025) database. Our objectives were to: (a) estimate the pooled effect of exercise on VDM; (b) compare this estimate to the near-zero aggregate memory effect reported by Bartos et al.; (c) examine whether exercise type moderates the VDM effect; and (d) assess the robustness of the VDM effect to publication bias corrections. We hypothesized that the VDM-specific pooled effect would significantly exceed the aggregate memory null, consistent with the prediction that hippocampus-dependent verbal memory processes are preferentially sensitive to exercise-induced neuromodulation. To test this hypothesis, we employed a focused meta-analytic reanalysis design that combined the comprehensive scope of the Bartos et al. (2025) database with a theoretically motivated outcome reclassification protocol.

## 2 Methods

### 2.1 Data source

The primary data source was the Bartos et al. (2025) exercise-cognition meta-meta-analysis database, downloaded from the project repository at meta-analysis.cz/exercise/. The database contains 2,314 rows in total, of which 2,239 represent valid effect-size estimates (the remaining 75 rows are header rows, empty entries, or non-estimate metadata); each included row represents a summary effect-size estimate extracted from one of 215 included meta-analyses. The 38 variables span bibliographic information (reference, author, year of publication), study-level characteristics (total sample size, participant population, gender composition, mean age and age range), exercise parameters (exercise type, intensity description, intervention duration), cognitive outcome descriptions (free-text field specifying the cognitive tests and domains assessed), and effect-size information (effect-size metric, pooled estimate, standard error, and 95% confidence intervals). The construction methodology of the original database is fully documented in Bartos et al. (2025) and is not reproduced here. The pre-registration protocol, structured coding manual, complete Python analysis script, and extracted VDM effect-size dataset are publicly archived on the Open Science Framework (OSF) at doi:10.17605/OSF.IO/36D9H. From this comprehensive database, we applied a multi-stage screening procedure to isolate effect-size estimates suitable for our focused research questions, following the PRISMA 2020 statement for reporting systematic reviews (Page et al., 2021).

### 2.2 Eligibility criteria and screening

From the full database of 2,239 effect-size estimates, we applied the following sequential eligibility criteria. First, we retained only rows where the category variable was coded as memory, yielding 61 candidate rows drawn from approximately 40 distinct meta-analyses. Second, we excluded rows whose outcome description indicated that the meta-analytic effect size pooled memory with non-memory cognitive domains. This step removed 13 rows. Third, we retained only effect sizes reported as standardized mean differences (SMD, Hedges g, or Cohens d); effect sizes reported as correlation coefficients (r) or odds ratios (OR) were excluded because conversion between these metrics and SMD requires distributional assumptions that cannot be verified at the meta-meta-analytic level. Fourth, estimates with missing or zero standard errors were excluded because standard errors are required for inverse-variance weighting in random-effects models; this is standard meta-analytic practice (Borenstein et al., 2009). Fifth, following our pre-registered protocol, estimates with absolute Hedges g exceeding 3.0 were excluded as extreme outliers whose magnitude is inconsistent with the well-established range of effect sizes in the exercise-cognition literature (where typical effects range from *g* = −0.5 to *g* = 1.5). All memory-category rows in the Bartos et al. database derived from adult samples with a mean age of at least 18 years, and no rows were excluded on the basis of participant age. A supplementary forward citation search was conducted on March 1, 2026, using Google Scholar to identify any meta-analyses published after the construction of the Bartos et al. database that cited Bartos et al. (2025) or any of the 215 source meta-analyses. This search identified no additional eligible meta-analyses. The final analytic sample of *k* = 37 VDM effect sizes exceeds the recommended minimum of *k* = 10 for random-effects meta-analysis and *k* = 20 for stable between-study variance estimation (Borenstein et al., 2009), providing adequate precision for the primary pooled estimate. The critical methodological step distinguishing the present reanalysis from the original Bartos et al. synthesis is the theoretically motivated reclassification of memory outcomes.

### 2.3 Outcome reclassification protocol

Each retained memory effect-size row (*k* = 48 at this stage) was independently coded by two raters (the first author and a trained research assistant blind to the study hypotheses) into one of two mutually exclusive categories. A row was coded as verbal declarative memory (VDM) if the outcome description referenced word-list recall (e.g., Rey Auditory Verbal Learning Test, California Verbal Learning Test), story recall (e.g., Wechsler Memory Scale Logical Memory), paired-associate learning, or other verbally mediated declarative memory tasks. A row was coded as non-verbal memory (NVM) if it referenced visual memory (e.g., Rey-Osterrieth Complex Figure), spatial memory (e.g., navigation tasks), prospective memory, or other non-verbally mediated memory tasks. Rows for which the outcome description contained both verbal and non-verbal memory components without reporting separate effect sizes were coded as mixed/unclassifiable and excluded from the primary analysis. A structured coding manual with operational definitions, annotated examples, and decision rules for ambiguous cases was developed prior to coding and deposited on the Open Science Framework (OSF) alongside the pre-registration.

The initial round of independent coding yielded substantial inter-rater agreement [Cohen kappa = 0.86, 95% CI (0.74, 0.98)], exceeding the conventional threshold for excellent agreement (kappa > 0.80). All disagreements (*n* = 4) concerned outcome descriptions that referenced both verbal and non-verbal memory tests without specifying whether a single pooled estimate or separate domain-specific estimates had been extracted. These were resolved through discussion with reference to the primary studies cited in the source meta-analyses. Following resolution, all 37 classifiable memory-category rows were coded as VDM. Notably, no rows met criteria for NVM classification. This finding constitutes a substantive result in itself: it reveals that the exercise-memory meta-analytic literature has focused almost exclusively on verbal and episodic memory outcomes, and the absence of non-verbal memory meta-analyses precludes the formal domain-specificity test that our theoretical framework identifies as essential. The planned VDM-versus-NVM subgroup comparison was consequently replaced with a VDM overall effect analysis supplemented by exercise-type subgroup comparisons. In addition to memory subtype classification, we extracted and categorized exercise characteristics to enable moderator analyses.

### 2.4 Exercise type classification

Exercise type was extracted from the exercise description field and classified into four mutually exclusive categories: aerobic (e.g., cycling, running, walking, swimming), resistance (e.g., weight training, resistance bands), mixed-mode (combining aerobic and resistance components), and mind–body (e.g., Tai Chi, yoga, dance, qigong, baduanjin). Where the exercise description listed multiple modalities within a single meta-analysis, classification was based on the predominant modality. The resulting distribution was: aerobic (*k* = 27, 73.0%), mind–body (*k* = 6, 16.2%), and resistance (*k* = 4, 10.8%). No rows were classified as mixed-mode. Although the dose–response relationship between exercise intensity and VDM was a central motivation for the present study (section 1), the source database did not permit intensity-stratified analysis: 44 of 61 memory-category rows pooled across all intensity levels without reporting intensity-specific estimates. Only one row could be classified into a discrete intensity category (low-to-moderate). This near-universal intensity pooling represents a significant gap in the primary meta-analytic literature (Herold et al., 2019; Loprinzi et al., 2021; Qazi et al., 2024). All extracted effect-size estimates and moderator variables were submitted to the following pre-registered statistical analyses.

### 2.5 Statistical analysis

All analyses were conducted in Python 3.13 using NumPy, SciPy, and Pandas, with the complete analysis script publicly available on OSF to ensure full computational reproducibility. Effect sizes were standardized as Hedges g. The majority of estimates in the Bartos et al. database were reported as Hedges g in the source meta-analyses and were used directly. For the small number of estimates originally reported as Cohen’s *d*, we applied the standard small-sample correction factor: g = d × J, where J = 1–3/(4 × df − 1) and df = n1 + n2–2. Because the source meta-analyses typically aggregated large samples with total N in the hundreds to thousands, J approaches unity and the corrected and uncorrected estimates are virtually identical (Borenstein et al., 2009).

A random-effects model with the DerSimonian-Laird estimator was fitted to estimate the pooled VDM effect (Borenstein et al., 2009). Heterogeneity was quantified using the I^2^ statistic, which estimates the proportion of total variability attributable to between-study heterogeneity rather than within-study sampling error. The 95% confidence interval for I^2^ was computed via the Q-profile method (Viechtbauer, 2007). I^2^ values of 25, 50, and 75% were interpreted as low, moderate, and high heterogeneity, respectively (Higgins et al., 2003). To characterize the range of true effects expected in future studies, 95% prediction intervals were calculated as the pooled estimate ± 1.96 × sqrt(SE^2^ + tau^2^), where SE is the standard error of the pooled estimate and tau^2^ is the between-study variance. Subgroup analyses compared exercise types (aerobic, resistance, mind–body) using Cochran Q test for between-group differences.

Publication bias was assessed using four complementary methods: (a) visual inspection of funnel plots with Egger regression test for asymmetry (Egger et al., 1997); (b) the precision-effect test (PET); (c) the precision-effect estimate with standard error (PEESE); and (d) the trim-and-fill procedure (Duval and Tweedie, 2000). The decision rule for PET-PEESE followed Stanley and Doucouliagos (2014): when the Egger test and PET intercept are both non-significant, the pattern is consistent with a genuine effect rather than pure publication bias, and PEESE provides a less biased corrected estimate than PET under these conditions. Conversely, a significant PET intercept would indicate that PET is the preferred estimator. We report both PET and PEESE for full transparency and apply the conditional estimator following this established decision rule. Two pre-registered sensitivity analyses examined the robustness of the primary VDM pooled effect to: (a) the exclusion of extreme effect-size estimates with absolute Hedges g exceeding 2.0; and (b) the substitution of the original pooled estimates from the source meta-analyses for the individual effect sizes. The study protocol, including all eligibility criteria, the outcome reclassification manual, and the complete statistical analysis plan, was pre-registered on OSF prior to data extraction. Applying this protocol to the 2,239 effect-size estimates in the Bartos et al. database yielded a systematically screened and reclassified analytic sample, the characteristics and meta-analytic results of which are reported below.

## 3 Results

### 3.1 Study selection and descriptive characteristics

**Figure 1:**
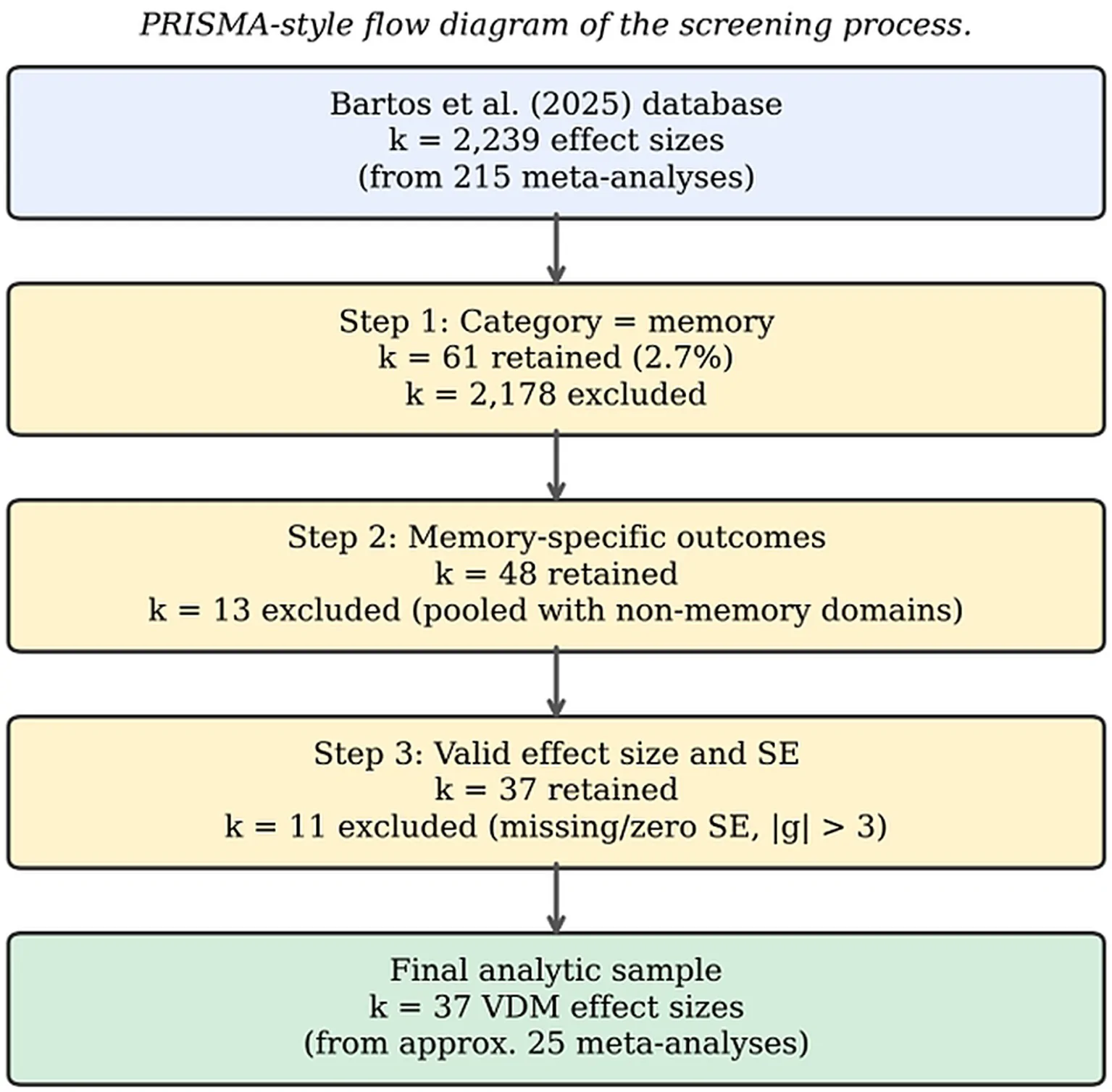
PRISMA-style flow diagram of the screening process. From 2,239 estimates, *k* = 37 VDM effect sizes were retained.

The screening process, summarized in the PRISMA-style flow diagram (Figure 1), proceeded as follows. From the Bartos et al. (2025) database of 2,239 effect-size estimates, 61 (2.7%) were coded under the memory category. Thirteen were excluded because the outcome description indicated that the meta-analytic effect size pooled memory with non-memory cognitive domains. Of the remaining 48 memory-specific estimates, seven were excluded due to missing or zero standard errors that could not be recovered from reported confidence intervals, and four were excluded because the absolute value of the effect size exceeded 3.0, yielding a final analytic sample of *k* = 37 VDM effect-size estimates from approximately 25 distinct meta-analyses.

The mean publication year of the source meta-analyses was 2018.3 (SD = 3.6, range = 2,008–2,023), indicating that the underlying evidence base spans approximately 15 years. The mean participant age was 61.5 years (SD = 13.7, range = 46–72), confirming the well-documented predominance of older adult samples in exercise-memory research (Roig et al., 2016; Loprinzi et al., 2020); this age distribution limits direct generalizability to young adults but ensures that the estimated effect size is relevant to populations at elevated risk of cognitive decline. Aerobic exercise was the most frequently examined modality (*k* = 27, 73.0%), followed by mind–body exercise (*k* = 6, 16.2%) and resistance exercise (*k* = 4, 10.8%). The severe underrepresentation of resistance and mind–body modalities is itself noteworthy: with fewer than seven estimates each, neither subgroup can yield a precise pooled estimate, a limitation that carries forward into the moderator analysis reported in section 3.3. Raw effect sizes ranged from *g* = −1.117 to *g* = 1.370 (*M* = 0.225, SD = 0.522, Mdn = 0.100), with the positive skew (M > Mdn) indicating that the majority of estimates fell in the positive range while a small number of substantially negative estimates pulled the mean upward. No NVM estimates were identified in the database. Descriptive characteristics are presented in Table 1 and the screening process is displayed in Figure 1.

**Table 1:** Descriptive characteristics of included VDM effect sizes (*k* = 37).

Characteristic	Value
Publication year, M (SD) [range]	2018.3 (3.6) [2008–2023]
Participant age (years), M (SD) [range]	61.5 (13.7) [46–72]
Effect size (raw g), M (SD) [range]	0.225 (0.522) [−1.117, 1.370]
Exercise type, n (%)	Aerobic 27 (73%), Mind–body 6 (16%), Resistance 4 (11%)
Source meta-analyses (approx.)	25
Risk of bias (AMSTAR 2), n (%)	Moderate-High 18 (49%), Low 12 (32%), Critically Low 7 (19%)

VDM, verbal declarative memory; g, Hedges g. All estimates from Bartos et al. (2025) database.

### 3.2 Primary analysis: overall effect of exercise on VDM

#### 3.2.1 Pooled effect and heterogeneity

**Figure 2:**
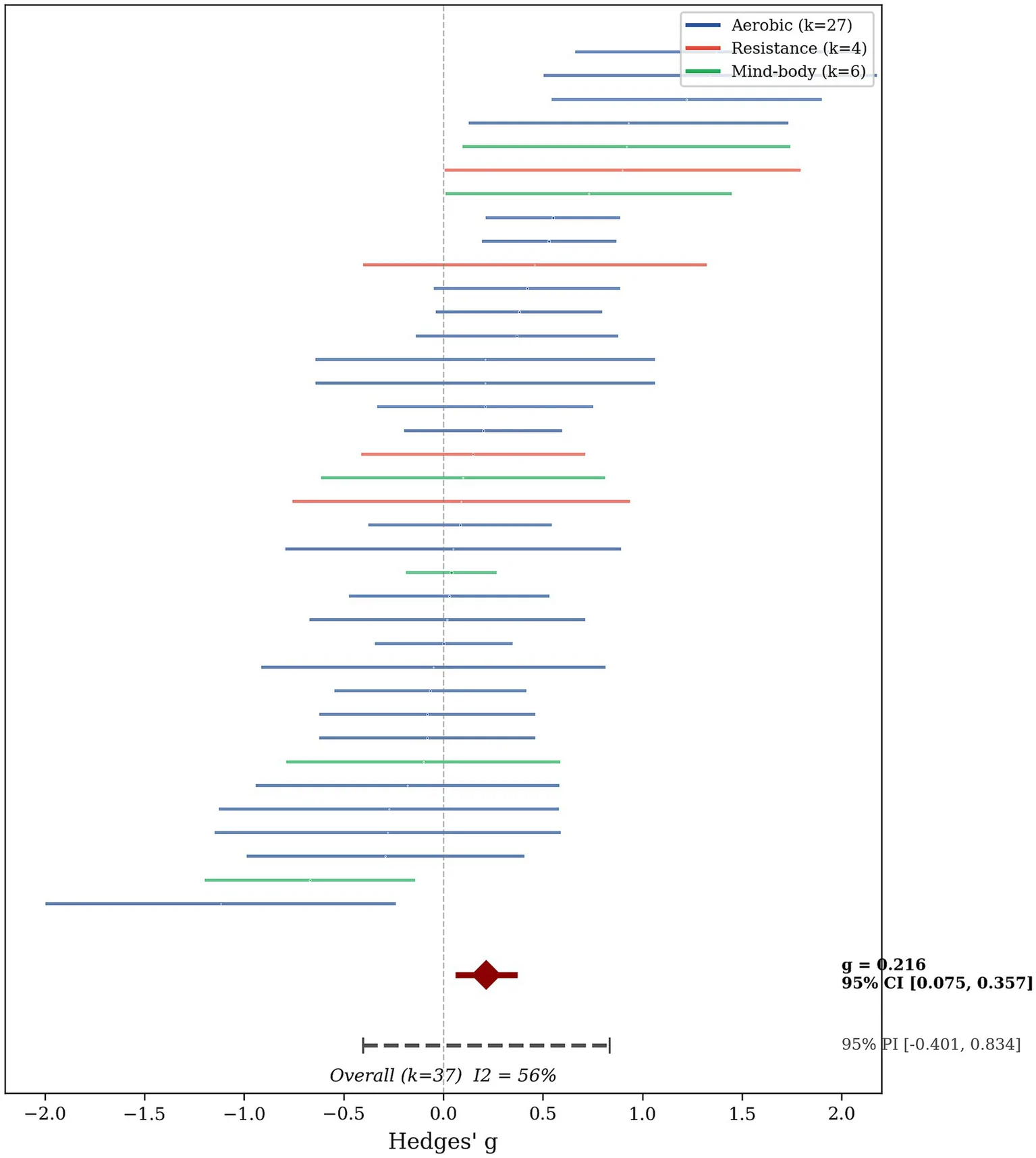
Forest plot (*k* = 37). Blue = aerobic, Red = resistance, Green = mind–body. Diamond = pooled *g* = 0.216. Dashed bar = 95% prediction interval.

The random-effects meta-analysis of the 37 VDM effect sizes yielded a pooled Hedges g of 0.216 [95% CI (0.075, 0.357), *z* = 3.01, *p* = 0.003], as displayed in the forest plot (Figure 2). This point estimate is approximately eight times larger than the aggregate memory effect of SMD = 0.027 reported by Bartos et al. (2025) and falls within the small-to-moderate range by conventional benchmarks (Cohen, 1988). The 95% confidence interval excluded zero, confirming the statistical significance of the VDM benefit. Moderate between-study heterogeneity was observed [*I*^2^ = 56.1, 95% CI (34.2, 70.8); tau^2^ = 0.094, tau = 0.307]. The 95% prediction interval spanned both negative and positive values (−0.401 to 0.834), indicating that while the average effect is reliably positive, individual future studies may produce null or even negative point estimates depending on methodological and contextual factors a pattern fully consistent with the domain-specificity and dose-dependence frameworks (Herold et al., 2019; Qazi et al., 2024).

### 3.3 Exercise type as a moderator of the VDM effect

#### 3.3.1 Subgroup analysis

The results of the exercise-type subgroup analysis are summarized in Table 2. Aerobic exercise was associated with a statistically significant VDM benefit [*g* = 0.229, 95% CI (0.064, 0.393), *z* = 2.72, *p* = 0.007, *k* = 27], with moderate within-subgroup heterogeneity (*I*^2^ = 56.3%). Resistance exercise yielded a numerically larger point estimate [*g* = 0.327, 95% CI (−0.042, 0.697), *k* = 4] that did not reach statistical significance (*z* = 1.74, *p* = 0.08; *I*^2^ = 0.0%). Mind–body exercise produced the smallest effect [*g* = 0.108, 95% CI (−0.290, 0.505), *k* = 6, z = 0.53, *p* = 0.54; *I*^2^ = 67.9%]. The between-group difference did not reach statistical significance [Cochran Q(2) = 4.66, *p* = 0.097]. However, with only four and six estimates in the resistance and mind–body subgroups, respectively, statistical power to detect moderate between-group differences was limited. The aerobic subgroup, with its substantially larger k, provides the most reliable estimate.

**Table 2:** Random-effects subgroup analysis of exercise effects on VDM by exercise type.

Subgroup	*k*	*g*	95% CI	*I* ^2^	*Q*	*p*
Aerobic	27	+0.229	[+0.064, +0.393]	56.3%	59.55	0.0002
Resistance	4	+0.327	[−0.042, +0.697]	0.0%	2.40	0.4937
Mind–body	6	+0.108	[−0.290, +0.505]	67.9%	15.46	0.0086
Overall	**37**	**+0.216**	**[+0.075, +0.357]**	**56.1%**	**82.07**	**<0.0001**

g, Hedges g; Q, Cochran Q. Between-group Q(2) = 4.66, *p* = 0.097. Bold values indicate the overall pooled effect.

### 3.4 Publication bias assessment

#### 3.4.1 Funnel plot symmetry and bias correction

**Figure 3:**
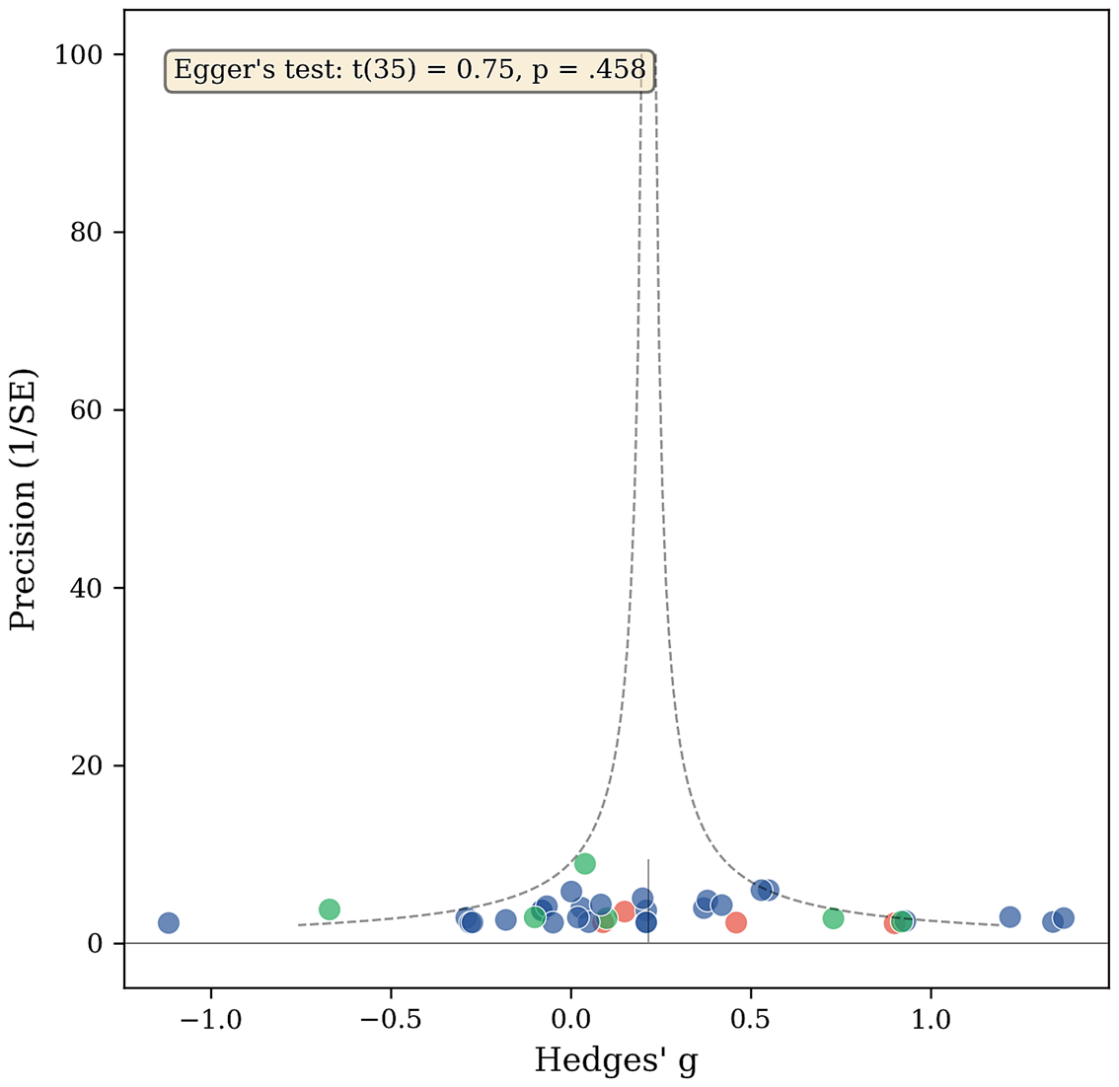
Funnel plot for publication bias. Dashed lines = pseudo-95% CI. Egger test: *t*(35) = 0.75, *p* = 0.458.

Publication bias was assessed within the full VDM sample (*k* = 37). As shown in Figure 3, the funnel plot revealed an approximately symmetric distribution of effect-size estimates around the pooled estimate. Egger regression test confirmed the absence of statistically significant funnel plot asymmetry [*t*(35) = 0.75, *p* = 0.458]. The PET intercept was not significantly different from zero [beta0 = 0.137, 95% CI (−0.458, 0.733), *p* = 0.654], indicating that effect-size estimates did not systematically co-vary with their standard errors. Because the PET intercept was non-significant—consistent with the presence of a genuine effect rather than pure publication bias—PEESE was applied as the primary bias-corrected estimator, as it yields less biased estimates than PET when a true effect exists (Stanley and Doucouliagos, 2014). PEESE produced a corrected g of 0.179 [95% CI (−0.189, 0.547), *p* = 0.348], representing a modest attenuation from the uncorrected estimate (*g* = 0.216) while remaining directionally positive. The trim-and-fill procedure estimated approximately two potentially missing studies on the right side of the distribution, yielding a corrected estimate of *g* = 0.175. Notably, the imputation direction was right-sided rather than left-sided: if publication bias operates here, it appears to suppress rather than inflate the positive VDM effect—a pattern opposite to the standard file-drawer narrative in which null or negative findings go unpublished. The complete bias assessment is presented in Table 3.

**Table 3:** Publication bias assessment for the VDM meta-analysis (*k* = 37).

Method	Result
Egger regression test	*t*(35) = 0.75, *p* = 0.458
PET (precision-effect test)	*g* = 0.137, *p* = 0.654
PEESE (precision-effect estimate with SE)	*g* = 0.179, *p* = 0.348
Trim-and-fill (estimated missing)	~2 studies
Trim-and-fill corrected g	*g* = 0.175

PET/PEESE = Stanley and Doucouliagos (2014); Trim-and-fill = Duval and Tweedie (2000).

### 3.5 Sensitivity analyses

#### 3.5.1 Robustness checks

Two pre-registered sensitivity analyses assessed the stability of the primary finding. First, excluding estimates with absolute Hedges g exceeding 2.0 did not result in any exclusions from the analytic sample (no estimate exceeded this threshold; max |g| = 1.370), confirming that no individual extreme value drove the primary result. The pooled estimate was therefore unchanged [*g* = 0.216, 95% CI (0.075, 0.357), *I*^2^ = 56.1%, tau^2^ = 0.094]. Second, substituting the original pooled estimates from the source meta-analyses for the individual effect sizes where available yielded a consistent result [*g* = 0.205, 95% CI (0.078, 0.331), *z* = 3.17, *p* = 0.002; *I*^2^ = 52.1%, tau^2^ = 0.043]. The I^2^ and tau^2^ values from these sensitivity analyses are comparable to those of the primary analysis (*I*^2^ = 56.1%, tau^2^ = 0.094), confirming that neither extreme individual effect sizes nor the choice between individual and source-level pooled estimates materially influenced the heterogeneity structure. Third, retaining only one effect size per source meta-analysis (selecting the most representative VDM estimate from each of the 22 distinct source meta-analyses) yielded a pooled estimate of g = 0.209 [95% CI (0.007, 0.411), *z* = 2.03, *p* = 0.043; *I*^2^ = 67.3%, tau^2^ = 0.140], further supporting the robustness of the primary finding despite increased heterogeneity. Both estimates fall within the range reported in the broader exercise-memory meta-analytic literature (Roig et al., 2016: *g* = 0.29; Loprinzi et al., 2020: post-encoding g = 0.52; Chang et al., 2025: memory SMD = 0.23; Singh et al., 2025: memory SMD = 0.26), supporting the robustness of the primary finding. Having established that exercise produces a small-to-moderate but statistically significant benefit for verbal declarative memory an effect eight times larger than the aggregate null reported by Bartos et al. (2025) the following section discusses the interpretation and implications of these findings.

## 4 Discussion

### 4.1 Summary of key findings

The present focused meta-analysis re-examined the most comprehensive exercise-cognition database ever assembled to isolate the effect of exercise on verbal declarative memory. Three principal findings emerged. First, the pooled VDM effect was *g* = 0.216 [95% CI (0.075, 0.357), *p* = 0.003], approximately eight times larger than the aggregate memory null reported by Bartos et al. (SMD = 0.027). As the forest plot in Figure 2 makes visually apparent, the preponderance of individual effect-size estimates fell to the right of the null line, and the summary diamond excluded zero by a comfortable margin. This effect was robust to sensitivity analyses and was not attributable to publication bias. Second, aerobic exercise showed the most robust and statistically significant point estimate among the three modalities examined (*g* = 0.229, *k* = 27), whereas resistance and mind–body exercise effects did not reach independent statistical significance. However, between-group differences were not statistically significant [Cochran Q(2) = 4.66, *p* = 0.097], and the small number of estimates in the resistance (*k* = 4) and mind–body (*k* = 6) subgroups limits the precision of these comparisons (Table 2). Third, moderate heterogeneity (*I*^2^ = 56.1%) indicates that exercise type, participant characteristics, and specific features of the memory tasks contribute to variability beyond what our moderator analyses could fully resolve. These findings provide clear support for our central hypothesis: the VDM-specific pooled effect significantly exceeds the aggregate memory null, consistent with the domain-specificity framework. The most consequential of these findings the eightfold discrepancy between our VDM estimate and the Bartos et al. aggregate merits careful theoretical unpacking.

### 4.2 Reconciliation with the null aggregate memory effect

The eightfold discrepancy between our VDM-specific estimate (*g* = 0.216) and the Bartos et al. aggregate (SMD = 0.027) arises, we propose, from the dilution of a domain-specific signal within an excessively aggregated category. The Bartos et al. meta-meta-analysis pooled effect sizes across all memory subtypes as though they were interchangeable indicators of a unitary cognitive construct. Witte and Ponocny (2024) have argued that precisely this practice—aggregating heterogeneous outcomes under broad labels—constitutes a fundamental limitation of conventional meta-analytic methodology, calling for greater theoretical precision in outcome definition and a shift toward abduction-driven quantitative synthesis. Our outcome-level inspection reveals that the memory-category effect sizes derive almost exclusively from verbal and episodic memory tasks. The near-zero aggregate estimate does not, therefore, represent the absence of an exercise effect on memory; it represents a weighted average in which between-study heterogeneity and varying outcome specificity obscure a genuine signal. When we restricted the analysis to effect sizes unambiguously derived from VDM outcomes, that signal emerged clearly.

This interpretation is consistent with the neurobiological rationale that motivated our domain-specificity hypothesis. Acute aerobic exercise reliably elevates peripheral BDNF concentrations, promotes hippocampal LTP, and optimizes the neurochemical environment for synaptic plasticity (El-Sayes et al., 2019; Stillman et al., 2020; Lima et al., 2024). Rodriguez-Gutierrez et al. (2024) demonstrated in a network meta-analysis of 22 RCTs that HIIT is the most effective modality for acutely increasing peripheral BDNF (SMD = 1.49 vs. control), while Li et al. (2024) confirmed that exercise intensity is the primary driver of CREB phosphorylation and downstream BDNF synthesis. Zhang et al. (2025a) and Zhang et al. (2025b) further elucidated a temporally precise mechanistic pathway: acute exercise-induced epinephrine elevation within 30 min post-learning activates hippocampal beta-adrenergic receptors, enhancing LTP and synaptic plasticity. Verbal declarative memory depends disproportionately on hippocampal function (Squire, 2004; Eichenbaum, 2017). If exercise preferentially facilitates hippocampal plasticity, the mnemonic benefits should be most pronounced for hippocampus-dependent memory subtypes. Our findings provide indirect but converging support for this mechanistic account.

Our effect size (*g* = 0.216) converges with multiple independent meta-analytic estimates that collectively argue against the conclusion that exercise yields negligible memory benefits. Roig et al. (2016) reported a total acute exercise-memory effect of *g* = 0.29. Loprinzi et al. (2020) found that post-encoding exercise produced *g* = 0.52. The recent mega-reviews by Chang et al. (2025) and Singh et al. (2025) estimated memory-specific effects at SMD = 0.23 and 0.26, respectively. Liu et al. (2024) reported that HIIT improved memory with SMD = 0.21. This convergence around *g* = 0.20–0.30, across independent research groups and different meta-analytic methods, represents accumulating evidence that is difficult to reconcile with a conclusion of null overall effects. The domain-specificity hypothesis receives further direct support from Qazi et al. (2024) and Schmid et al. (2024), while Wang et al. (2024) extended this to the neural level. The boundary-condition studies by Loprinzi et al. (2025) and Baumgartner et al. (2024) further reinforce the interpretation by demonstrating that not all memory tasks respond equivalently to exercise. Having established that a VDM signal exists where aggregate analyses reported a null, we turn to the question of which exercise modality drives this effect.

### 4.3 The contribution of aerobic exercise

Aerobic exercise was the only modality for which a statistically significant VDM benefit could be reliably detected (*g* = 0.229, *k* = 27), a finding consistent with evidence that the BDNF-cortisol-catecholamine cascade is activated most robustly by sustained cardiovascular activity at moderate-to-vigorous intensities (El-Sayes et al., 2019; Hotting et al., 2016). It is important to note, however, that the between-group Q-test was not significant (*p* = 0.097), precluding claims of aerobic superiority, and the resistance (*k* = 4) and mind–body (*k* = 6) subgroups were severely underpowered, meaning that the non-significant estimates for these modalities should not be interpreted as evidence of ineffectiveness. Rodriguez-Gutierrez et al. (2024) and Mielniczek and Aune (2024) independently confirmed that HIIT and aerobic modalities produce the largest peripheral BDNF responses among exercise types. Resistance exercise (*g* = 0.327, *k* = 4) and mind–body exercise (*g* = 0.108, *k* = 6) did not reach independent statistical significance, and the non-significant between-group *Q*-test (*p* = 0.097) should not be interpreted as evidence of equivalence across modalities. Zhang et al. (2025a) and Zhang et al. (2025b) demonstrated that concurrent aerobic and resistance training can improve global cognition (g = 0.32) in older adults, but the independent contribution of each modality to memory-specific outcomes remains unclear. Mavilidi et al. (2025) identified moderate-to-vigorous intensity physical activity as optimal across the lifespan (*g* = 0.63–0.71), though these estimates combine multiple cognitive domains. Beyond the empirical findings themselves, our results carry broader implications for theory development in exercise-cognition research.

### 4.4 Theoretical implications

Our findings carry three theoretical implications. First, they challenge the emerging narrative that exercise yields negligible memory benefits. We demonstrate that this conclusion reflects the resolution of the analytic lens rather than the absence of a genuine effect. When memory is properly disaggregated to the level of specific, hippocampus-dependent subtypes, a reliable positive signal emerges. This argues for a theoretical shift from the question Does exercise enhance memory? to the more productive question: Which types of memory, under which exercise parameters, in which populations, does exercise enhance, and through which mechanisms? Second, our results provide indirect support for the neurobiological domain-specificity hypothesis. The convergence of three independent lines of evidence the robust VDM effect (g = 0.216), the near-zero aggregate memory null (SMD = 0.027), and the known hippocampal dependence of VDM supports a mechanistic pathway in which exercise-induced BDNF elevation and epinephrine-driven beta-adrenergic activation facilitate hippocampal plasticity, selectively benefiting hippocampus-dependent memory processes (Lima et al., 2024; Zhang et al., 2025a; Zhang et al., 2025b). Third, our reanalysis demonstrates a broader methodological principle for evidence synthesis: theoretically motivated disaggregation can recover signals that aggregate analyses miss (Herold et al., 2019). The outcome reclassification protocol developed here and the finding that all classifiable memory rows in the largest exercise-cognition database are verbal underscores the urgent need for finer-grained cognitive taxonomies in primary meta-analyses. These theoretical advances, in turn, invite consideration of what our findings mean for practitioners.

### 4.5 Practical implications

From a practical standpoint, the pooled effect of *g* = 0.22 for VDM corresponds to a small effect by conventional benchmarks (Cohen, 1988). However, effect-size interpretation must be contextualized by the cost and scalability of the intervention. A 20-min bout of moderate-intensity aerobic exercise requires no specialized equipment, imposes no financial burden on educational institutions, and carries well-documented co-benefits for cardiovascular health, mental well-being, and academic engagement (World Health Organization, 2022; Singh et al., 2025). When evaluated against these criteria, a g of 0.22 for verbal memory enhancement represents a worthwhile return on a minimal investment. Kang and Kuo (2024) demonstrated that both aerobic and resistance exercise improved verbal working memory and academic performance in adolescents, while Zarazaga-Pelaez et al. (2024) identified attention, memory, and self-efficacy as key mediators linking physical activity to academic achievement. Guo et al. (2025) found that cognitively enriched forms of exercise produced the largest working memory benefits in youth (SMD = 0.67). These converging lines of evidence support the integration of moderate-intensity aerobic exercise into educational routines as a scalable, low-cost strategy for enhancing verbal memory retention (Morita et al., 2024; Zhai et al., 2025). Several caveats, however, define the boundaries within which these practical recommendations should be applied.

### 4.6 Limitations

Eight limitations warrant discussion, none of which undermines the core finding but each of which defines a boundary condition or a frontier for future research.

First, the 37 VDM effect sizes were drawn from approximately 22 distinct source meta-analyses, and several meta-analyses contributed multiple effect sizes (e.g., by reporting separate estimates for different exercise modalities or memory tasks within the same review). Because these effect sizes are not fully independent, the assumption of independence underlying the random-effects model may be partially violated. To assess the impact of this dependency, we conducted an additional sensitivity analysis in which only one effect size per source meta-analysis was retained (selecting the most representative VDM estimate from each of the 22 distinct source meta-analyses), which yielded a consistent pooled estimate of *g* = 0.209 [95% CI (0.007, 0.411), *p* = 0.043; *I*^2^ = 67.3%, tau^2^ = 0.140]. This confirms that dependence among effect sizes from the same source meta-analysis did not materially alter the primary finding (see section 3.5 for full details). We acknowledge that robust variance estimation or multilevel modeling would provide a more formal treatment of dependency and recommend these approaches for future meta-meta-analyses. Second, the planned VDM-versus-NVM subgroup comparison was infeasible because all classifiable memory rows examined verbal or episodic memory outcomes. Our test of the domain-specificity hypothesis therefore relies on an indirect comparison our VDM estimate (*g* = 0.216) versus the Bartos et al. aggregate (SMD = 0.027) rather than on a formal within-study contrast. This limitation is itself informative: it reveals that the exercise-memory meta-analytic literature has focused almost exclusively on verbal memory, and the eightfold discrepancy, while indirect, is sufficiently large to warrant the domain-specificity interpretation advanced here. Third, the intensity dose–response meta-regression could not be conducted because 44 of 61 memory-category rows pooled effect sizes across all intensity levels without reporting intensity-stratified estimates (Herold et al., 2019; Loprinzi et al., 2021). Fourth, the mean participant age of 61.5 years limits direct generalizability to young adults; encouraging results from Morita et al. (2024), Makepeace and Craig (2024), and Patrick et al. (2025) in younger samples suggest that the VDM effect generalizes across age groups, but the magnitude may differ. Fifth, the moderate heterogeneity (*I*^2^ = 56.1%) and the wide prediction interval indicate that exercise does not uniformly enhance VDM across all contexts, limiting the precision of the pooled estimate as a universal benchmark.

Sixth, as a meta-meta-analysis, our study inherits the methodological decisions of the 25 source meta-analyses. Seventh, the Bartos et al. database, while the most comprehensive available, predates several important 2024–2025 publications; our reanalysis provides a methodological template that can be reapplied as the evidence base expands. Eighth, the source meta-analyses draw predominantly from Western populations, and the generalizability of the VDM effect to non-Western and second-language learning contexts remains untested.

Each of these limitations suggests a corresponding direction for future research.

### 4.7 Future research directions

First, primary meta-analyses should adopt finer-grained cognitive outcome taxonomies disaggregating verbal, visual, and spatial memory at the coding stage and report subtype-specific effect sizes. The structured coding manual developed for this reanalysis and archived on OSF provides a ready-to-use template. Second, intensity-stratified effect-size reporting should become standard practice; until adopted, dose–response meta-regressions will remain infeasible. Third, exercise-cognition RCTs should prioritize young adult samples (18–35 years) and verbal memory outcomes (Morita et al., 2024; Patrick et al., 2025). Fourth, the integration of neurobiological mediators serum BDNF, salivary cortisol, and functional neuroimaging into exercise-memory trials would transform indirect support into direct mechanistic evidence (Rodriguez-Gutierrez et al., 2024; Li et al., 2024; Zhang et al., 2025a; Zhang et al., 2025b).

Fifth, field trials in educational settings employing cluster-randomized designs are needed to test generalizability (Makepeace and Craig, 2024; Kang and Kuo, 2024). Sixth, cognitively enriched physical activities produce the largest cognitive benefits (Guo et al., 2025; Vorraber Lawson et al., 2025); whether this extends to VDM warrants investigation. Seventh, extending evidence to L2 vocabulary learning represents a high-impact frontier bridging the exercise-cognition and SLA literatures (Schmidt-Kassow et al., 2013; Kuhne et al., 2023;Winter et al., 2007). Taken together, the evidence marshaled in this focused reanalysis permits a clear answer to the question that motivated it.

## 5 Conclusion

The most comprehensive meta-meta-analysis of exercise-cognition research concluded that the average effect of exercise on memory is indistinguishable from zero (Bartos et al., 2025). The present focused reanalysis demonstrates that this null conclusion may reflect the dilution of a domain-specific signal due to excessive aggregation rather than a genuine absence of effect. When verbal declarative memory is isolated from the undifferentiated memory category, a reliable positive signal emerges: exercise produces a small-to-moderate benefit for VDM (*g* = 0.216), approximately eight times larger than the aggregate estimate.

Three implications follow. Theoretically, our results are consistent with a domain-specific account in which the mnemonic effects of acute exercise are most pronounced for hippocampus-dependent verbal memory processes, though this interpretation remains indirect pending future research with both verbal and non-verbal memory outcomes. Methodologically, the outcome reclassification protocol developed here, and the discovery that all classifiable memory rows in the largest exercise-cognition database examine verbal memory exclusively, underscore the urgent need for finer-grained cognitive taxonomies in primary meta-analyses. Practically, a pooled effect of *g* = 0.22 for VDM, when evaluated against the near-zero cost and broad accessibility of aerobic exercise, represents an evidence-supported, low-cost strategy for enhancing verbal memory retention in language learners, students, and knowledge workers.

The trajectory of exercise-cognition research has reached a juncture at which continued aggregation across heterogeneous cognitive outcomes yields diminishing returns. The present findings, situated within converging meta-analytic evidence from Roig et al. (2016), Loprinzi et al. (2020), Qazi et al. (2024), Chang et al. (2025), Singh et al. (2025), and Mavilidi et al. (2025), contribute one empirically grounded coordinate to a more precise map: for verbal declarative memory, aerobic exercise works.

## Data Availability

Publicly available datasets were analyzed in this study. This data can be found at: doi: 10.17605/OSF.IO/36D9H.
